# Response to COVID-19 in the Central African Republic: Coping Strategies Combined With China’s Experience

**DOI:** 10.3389/ijph.2022.1604344

**Published:** 2022-04-25

**Authors:** Qiheng Gou, Fubin Zhu, Keqi Xie, Yiping Li, Yuxin Xie

**Affiliations:** ^1^ Department of Head and Neck Oncology, Cancer Center, West China Hospital, Sichuan University, Chengdu, China; ^2^ Department of Anesthesiology, Mianyang Central Hospital, Mianyang, China; ^3^ Department of Public Health, College of Veterinary Medicine, Cornell University, New York, NY, United States; ^4^ Laboratory of Molecular Diagnosis of Cancer, Clinical Research Center for Breast, West China Hospital, Sichuan University, Chengdu, China

**Keywords:** COVID-19, China, Central African Republic, response, prevention and control

## Abstract

**Objectives:** The weak health system, domestic political unrest, poverty, and many other factors in the Central African Republic (CAR) have left the country underprepared for the COVID-19 pandemic, resulting in a greater health threat to the entire country. Rapid measures must therefore be taken to prevent the further spread of COVID-19.

**Methods:** This work encompassed a review of relevant literature. We aim to analyze how far Chinese COVID measures can be transferred to the context of the CAR.

**Results:** We argue that the measure that the CAR can learn from China’s success is the involvement of community workers and that greater investment in this model may be the optimal solution. Help from the international community is urgently needed.

**Conclusion:** The CAR can benefit from China’s successful experience in fighting the epidemic, but the disparity in the combined power of the two countries does not allow for simple replication of China’s strategy.

## Introduction

The new coronavirus disease (COVID-19) is a highly contagious respiratory disease caused by a new coronavirus initially reported in Wuhan in December 2019 [1]. It has been disseminated worldwide since the first quarter of 2020, posing significant challenges to the economies and health systems of many countries. For developing countries, the blows may even be fatal. Since the first case of COVID-19 was reported in Egypt in February 2020, COVID-19 has been reported in other African countries successively, most of which were low-income countries, such as the Central African Republic (CAR) [2]. The preparedness of a country such as CAR, which is affected by unrest, poverty, and a high prevalence of infectious diseases such as malaria and acquired immunodeficiency syndrome (AIDS), has become increasingly difficult. The emergence of new outbreaks of infectious diseases in this vulnerable region could lead to further collapse of the economy and health structure. COVID-19 prevention measures have been adopted globally but do not always take into account the economic, political, and sociocultural differences between countries and regions. We aim to analyze the extent to which Chinese measures to combat COVID-19 are transferable to the CAR context and why the CAR-specific context requires international help and therefore cannot simply replicate the Chinese strategy.

## Methods

Two experienced medical librarians conducted a series of database searches designed to identify literature on prevention and treatment experience of COVID-19 between CAR and China. These official websites including the United Nations, the International Committee of the Red Cross (ICRC) and governments were chosen to provide a complementary information. Database-specific controlled vocabulary terms including “Coronavirus,” “Coronavirus Infections,” “Central African Republic,” and “China” were used in combination with relevant text words, including specific terms for COVID-19 and SARS-COV-2 and terms representing concepts related to national conditions, prevention measures, strategies and schemes. The searches were conducted between December 30, 2019 and February 20, 2022.

## Results

### Status Quo and Predicaments of the Central African Republic

The Central African Republic is located in the middle of the African continent and shares borders with Sudan, South Sudan, the Democratic Republic of Congo (DRC), the Republic of Congo, Cameroon, and Chad. It was invaded by France in 1885, became a French colony in 1891, and confirmed its declaration of independence in 1960. After independence, the CAR has endured a prolonged military conflict.

Politically, in recent years, different armed groups have been expanding their spheres of influence and controlling the country’s rich mineral resources through several rebellions, further contributing to violence and human rights abuses and causing serious political instability [3]. Economically, the CAR is a country with a population of only 4.8 million, of which more than 70% of the population are living in multidimensional poverty. Up to 70% of workers are in precarious employment, and the informal economy (e.g., street vendors, informal sector farmers, home-based workers) contributes 50–80% of gross national income (GDP) [4]. The country’s GDP per capita in 2020 was only US$510. The agriculture, forestry, and livestock sectors account for more than 30% of GDP, and exports of goods and services account for up to 34% of GDP, thanks to the abundance of natural resources [5].

From sleeping sickness to AIDS to monkey pox, the CAR has always been a region where diseases emerge and are prevalent. It is also a hotspot for research on emerging diseases. Since the French colonial period, the country has received considerable support in epidemiological surveillance and has a well-established disease surveillance system [6]. Despite a good track record in disease surveillance, performance in primary health care is not as good. According to limited data reported by the World Bank, the CAR had 1 hospital bed per 1,000 citizens in 2011, only 1 nurse or midwife per 5,000,000 inhabitants in 2015, and an even greater shortage of doctors, with 1 internal medicine doctor per 14,000 [7]. The country’s civil war has forced qualified health practitioners to flee violence-prone areas, while the destruction of basic health facilities by the war had a devastating effect [8]. The provision of primary health care is largely dependent on the country’s nongovernmental organizations (NGOs) and international charities. According to the 2020 human development report, the human development index of the CAR in 2019 ranked 188th out of 189 countries in the world, which was only 0.397. It was significantly lower than the world average of 0.737 [9]. In addition, citizens of this country suffer from many challenges, such as a high disease burden, high neonatal mortality, hunger, and lack of access to safe drinking water [10]. Studies have shown that the CAR has the worst health situation [11].

On March 14, 2020, the CAR announced the first confirmed case of COVID-19. Although many positive reactions were taken by local authorities after the first carrier of the novel coronavirus was identified, COVID-19 was still not well controlled, and the number of positive COVID-19 cases continued to increase. Initially, most of the new cases were imported from outside the country; however, as capacities of screening and testing expanded, many local cases were gradually detected.

Rapid urbanization in sub-Saharan Africa has resulted in approximately 60% of urban residents living in slums or informal settlements [12]. Urbanization has led to more densely populated areas, and studies have shown that population density is a risk factor for COVID-19 transmission [13]. However, the prevalence of rural inhabitants increases the likelihood of staggered outbreaks, the peak of which comes later than in urban areas [14]. Due to Africa’s special population pyramid, climatic conditions, and other factors, fortunately, the incidence is relatively low compared to other continents [15]. In the CAR, by June 2020, local cases had surpassed imported cases from outside the country [16]. According to data from John Hopkins University, as of March 10, 2021, a total of 5,023 confirmed COVID-19 cases and 63 deaths have been reported in the CAR [2].

The lack of health resources, decades of civil unrest, and limited financial capacity are all multiple factors that are stumbling blocks to stopping the spread of the COVID-19 pandemic in the CAR. If the necessary measures are not taken to organize the spread of the disease, the results could be catastrophic. In contrast to Africa, China, the first country to report cases of COVID-19, has done a good job of containing the epidemic. In terms of national background, medical capacity, and political system, the CAR is very different from China. However, it is undeniable that China, as one of the most populous countries in the world, despite being the second-largest economy in the world, still needs many low-cost strategies to provide security for its population during the epidemic. Can the CAR, therefore, learn from the successful experience of China?

### Rapid Response to COVID-19 in China

In December 2019, 27 cases of unexplained pneumonia were reported in Wuhan, China, and the pathogen was identified as SARS-COV-2 in January 2020 [17]. After that, the government launched the national emergency response and took a series of aggressive and tough response measures, such as a lockdown of the city of Wuhan and other cities in Hubei Province. Studies have shown that the strict measures to restrict population movement in Wuhan and Hubei Province mitigated COVID-19 transmission, effectively controlling the source of infection and creating the conditions for further effective control of COVID-19 [18, 19]. Meanwhile, during the pandemic, authorities quickly developed diagnosis and treatment protocols for patients with COVID-19 and actively launched epidemiological investigations and laboratory tests [20]. The confirmed cases were treated in isolation at designated points, and the contacts were also under close medical observation during the incubation period. China established a large number of Fangcang shelter hospitals to provide patients with basic medical care and centralized isolation and observation. China’s Fangcang shelter hospitals are large temporary hospitals converted through public places such as gymnasiums and convention centers, which can provide basic medical care and centralized isolation and observation for patients with COVID-19 [21]. These actions have controlled the source of infection and interrupted the transmission of COVID-19 to some extent.

Although China is the second-largest economy in the world, there are still many remote and impoverished areas. This required the participation of local community workers and volunteers. Heavy tasks, such as daily temperature measurement, distribution of necessities, transfer of patients, and disinfection of public places, have been performed to prevent further transmissions. The government has also taken a series of proactive measures to ensure the physical and psychological well-being of community workers [22].

In summary, China’s success can be attributed to three factors. First, early detection capacity, a well-developed reporting system, and the unified deployment of health system practitioners by the authorities have all created the conditions for a rapid response. Second, the extensive involvement of community health workers and the long-term commitment to primary health care underpinned the health system’s ability to perform. Third, personal hygiene, social distancing, and blockades were necessary to organize the spread of the epidemic.

### Is China a Suitable Role Model for the CAR?

The Chinese measures to combat COVID-19 can only be effective to a limited extent in the CAR. Similar to China, the CAR generally has a good detection system but lacks personnel which inhibits the efficiency of the measures. Of course, the CAR has taken the necessary measures, but it did not work out as well as in China. There are several main reasons for this. First, as mentioned earlier, the CAR has a complete surveillance system for infectious diseases, which is an advantage for it. In January 2020, a treatment center for COVID-19 was already in operation. The strategy at the outset was to test, isolate, treat and track contacts and to screen people in transit. Subsequently, as the number of cases in the community increased and testing capacity was limited, testing began only for people with fever and/or COVID-19-like symptoms [16].

Second, although previous studies have demonstrated the important role of community health workers in rapid malaria diagnosis and treatment, the number and training of community health workers remain problematic due to ongoing conflict [3]. The WHO, the United Nations and the International Committee of the Red Cross (ICRC) have taken active steps. After the arrival of the COVID-Crisis in CAR, the ICRC trained community representatives on COVID-19 prevention measures. They also learned how to make simple hand-washing stations using local materials (four wooden sticks and a water container). With the support of the CAR Ministry of Health and the World Health Organization, more than one millione people were informed about COVID-19. As the conflict between government forces and a coalition of armed groups continues in several areas of the country, forcing the displacement of more than 180,000 people, and as adequate security for international aid workers and community workers is lacking, this makes relief supports difficult to implement [16, 23–25]. Even though the CAR has also employed community workers, this proved inefficient, possibly because of straining community livelihoods and services and understaffing [25]. Third, purchasing food and daily necessities is a challenge for maintaining social distance as well as isolation measures. The increased poverty and difficulty in food security brought about by the public transportation embargo cannot be ignored either. Moreover, populations in the CAR place a high value on collective culture. In collectivism, social relations are expressed in interdependence, maintenance and adherence to traditional customs, and the organization of collective life [26]. Social alienation and self-isolation can lead to social isolation and stigmatization, and it is important to have the support of loved ones (e.g., visits from family and friends during illness) [27].

In summary, the CAR cannot exactly replicate China’s success measures because of its limited state capacity. Currently, trained workers, good infrastructure, laboratories with testing capabilities, medical equipment, and drugs are all necessary. However, the CAR lacks a robust health financing system. Government spending accounts for only 13.6% of the share of health expenditures. It relies more on health development assistance, which accounts for 50%, and the lack of national medical supplies and personnel prevents it from conducting widespread detection [27]. A model that involves community health workers may be an effective solution. This model requires less human and material investment while achieving results faster, which is what the CAR needs. In addition, the CAR urgently needs material and human assistance from regional partners and the international community.

### International Community Assistance

This increase in medical personnel will facilitate effective surveillance of COVID-19 transmission patterns by improving detection capacity and expanding detection coverage, which will also provide more people with access to health care measures to stop the spread of the virus. The presence of United Nations peacekeeping troops is also necessary to maintain regional stability and protect vulnerable groups such as medical staff and patients in medical institutions. Assistance in the form of personal protective equipment, medicines, vaccines, and nucleic acid testing kits would help expand testing and improve treatment capacity [27]. In addition, assistance for basic needs supply is essential in a country with food insecurity and political instability. A further increase in medical personnel would increase the capacity of delivering curative and preventive health services.

According to the Development Assistance Committee of the Organization for Economic Cooperation and Development, the main sources of development assistance to CAR in 2019 were the World Bank, European Union Institutions, the United States, the United Nations Children’s Fund, and Germany. They provide US$169.2, US$115.5, US$104.0, US$59.26, and US$54.84 million, respectively. Approximately 108.9 million of them were used for the main aid purpose of the Emergency Response [28]. On March 16, 2020, the Ma and Alibaba Public Welfare Foundation donated 100,000 masks, 1,000 protective suits, 1,000 protective masks, and 20,000 test kits and committed to working with medical institutions in African countries to provide online training materials for the clinical treatment of the new coronavirus [29]. In addition, the WHO also called for US$675 million to support the most vulnerable African countries. Personal protective equipment was provided to African countries, as well as an online training course on COVID-19 [30]. The Chinese government also indicated on March 3, 2021, that it would assist the Central African Republic with a shipment of the new crown vaccine but did not report mentioning its exact quantity [31].

This may not be enough, and we call on more international aid, nongovernmental organizations, and agencies to join forces and take action to help the CAR facing this global public health crisis. We also hope that more long-term sustainable investments will be injected into the CAR to improve the entire health care system so that in the future they can respond early when epidemics occur and avoid their further spread. However, further in-depth studies are required to answer the question of the exact amount of supplies and the number of personnel they need.

## Discussion

The CAR has made considerable efforts to combat COVID-19, including improving detection capacity, strengthening preventive measures, and admitting confirmed cases whenever possible. However, as the number of confirmed cases is increasing, it is still not fully prepared for a COVID-19 pandemic. China, the first country to report cases of COVID-19, has done a good job of containing the epidemic. To some extent, the CAR can benefit from China’s successful experience in fighting the epidemic, but the disparity in the combined power of the two countries does not allow for simple replication of China’s strategy. We argue that the measure that the CAR can learn from China’s success is the involvement of community workers and that greater investment in this model may be the optimal solution. Help from the international community is urgently needed. Several countries and international organizations have already acted to help this troubled country through this difficult time. While such donor-dependent resources are not unlimited, allowing COVID-19 to run rampant in the CAR could exacerbate ongoing civil unrest and conflict and even threaten other parts of the world. We hereby call for more action from international, regional, and relevant countries in a position to do so, including the United Nations and the African Union.

## Abbreviations

COVID-19, new coronavirus disease; CAR, Central African Republic; WHO, World Health Organization; SARS-CoV, severe acute respiratory syndrome coronavirus; DRC, Democratic Republic of Congo; AIDS, acquired immunodeficiency syndrome; HAQ, healthcare access and quality; ICRC, Committee of the Red Cross.
